# Evaluation of Tools to Assess Symptoms and Symptom Severity in Patients With Medically Unexplained Physical Symptoms: A Systematic Review and Narrative Synthesis

**DOI:** 10.7759/cureus.56204

**Published:** 2024-03-15

**Authors:** Ananta Gopal Kuanar Baboo, Piyush Ranjan, Tanveer Kaur, Nandini Rawat, Siddharth Sarkar, Gaurishanker Kaloiya, Amandeep Singh, Natesh Babu, Upendra Baitha, Bindu Prakash

**Affiliations:** 1 Yoga, Swami Vivekananda Yoga Anusandhana Samsthana, Bangalore, IND; 2 Medicine, All India Institute of Medical Sciences, New Delhi, IND; 3 Psychiatry, All India Institute of Medical Sciences, New Delhi, IND; 4 Clinical Psychology, All India Institute of Medical Sciences, New Delhi, IND

**Keywords:** medically unexplained symptoms, mus, psychometric properties, validity, reliability, mups

## Abstract

A substantial portion of patients presenting to healthcare settings exhibit physical symptoms lacking clear, demonstrable organic causes. Accurate assessment of symptom severity is crucial for documenting outcomes and establishing treatment efficacy. This systematic review and narrative synthesis aims to provide researchers with insights into available and validated tools for assessing medically unexplained physical symptoms (MUPS).

It involved comprehensive searches across electronic databases, including PubMed, Wiley, and Cochrane, adhering to PRISMA and COSMIN guidelines. The study comprised two phases: Phase 1 systematically reviewed tools for assessing MUPS symptoms and severity, while Phase 2 conducted a narrative synthesis of their measurement properties, focusing on validity and reliability.

Out of 14,459 records, 191 articles were identified, leading to the recognition of 16 validated tools for assessing MUPS symptoms and severity. Most tools demonstrated excellent internal consistency and structural validity. However, the majority lacked cross-cultural validity.

The choice of tools for the assessment of MUPS will assist clinicians and researchers in determining the severity of MUPS and developing a tailored treatment plan to improve the physical and psychological functioning of these patients.

## Introduction and background

A significant number of patients in healthcare settings present with physical symptoms such as fatigue, pain, gastrointestinal issues, and others that have no clear organic cause [1]. Such symptoms are known as medically unexplained physical symptoms (MUPS). While some patients have self-limiting mild symptoms, a significant number of cases develop persistent and severe symptoms over time, leading to significant physical and psychological dysfunction [2].

Assessing the severity of symptoms in MUPS patients is vital for their management and research. Several tools have been used worldwide to assess symptoms and symptom severity among MUPS patients. Therefore, knowledge of these tools is crucial for doctors of different specialties treating MUPS patients.

To date, there has been no systematic review that identifies the instruments to assess MUPS patient symptoms and their severity, along with a synthesis of their methodological quality. This systematic review aims to provide researchers with a bird's-eye view of the available and easy-to-use tools. Identifying these tools would assist clinicians and researchers dealing with MUPS in gauging the severity of the problem and developing customized treatment plans to improve the physical and psychological functioning of these patients.

## Review

Methods

The study's methodology was registered in PROSPERO (ID-363209). The draft of the manuscript followed the Preferred Reporting Items for Systematic Reviews and Meta-Analyses (PRISMA) statement [3] and the COSMIN checklist [4]. The methodology consisted of two phases. Phase 1 included a systematic review of the tools to assess the symptoms and symptom severity of patients with MUPS, followed by Phase 2, which consisted of a narrative synthesis of the measurement properties of the identified tools (validity and reliability).

Symptoms were defined as the physical features for which the patients were seeking help from the practitioner, whereas, symptom severity is defined as the impact of these symptoms on an individual's physical functioning, mental functioning, and general well-being.

Phase 1: systematic identification of the instruments to measure symptom profile in patients with MUPS

Literature Search

An electronic search on three databases, PubMed, Cochrane, and Wiley, was performed by the authors (AK and NR) to search for the relevant articles published between January 2000 and January 2022. A search string “(MUPS OR MUS OR ‘Unexplained physical symptoms’ OR ‘medically unexplained physical symptoms’) AND (questionnaire OR scale OR tool OR instrument)” was used for searching for relevant indexing terms. We also searched for gray literature on the Open Grey database. A lateral search was also carried out using a reference list and cited literature for primary articles.

Inclusion and Exclusion Criteria

The inclusion criteria include studies measuring the symptoms and/or severity of symptoms in patients with MUPS using an assessment. Out of 30 tools identified, only 16 validated tools were included in this review, as the remaining 14 tools were either in some other language or information regarding the validation process was not accessible, with a population >18-60 years of age.

The exclusion criteria include papers that did not mention the outcomes and studies that did not mention the measures used to assess the symptoms and/or severity of symptoms in MUPS patients or used non-validated tools or questionnaires.

Study Selection

Two independent reviewers (TK and AK) carried out the title and abstract screening of the articles selected through the keyword search. The remaining articles were then read completely and were categorized as included (meeting inclusion criteria), excluded (not meeting inclusion criteria), and unclear (disagreement after independent review). The disagreement was resolved by third-party adjudication (PR). The selected articles were then included in the PRISMA flowchart for narrative synthesis.

Data Extraction and Synthesis

Two authors (AB and NR) independently extracted data from the finalized articles. Details such as the name of the tool, number of items, domain assessed, rating scale, time frame, target population, and availability in multiple languages were extracted. The correctness and completeness of the data were done by authors (PR and TK). In case of any discrepancy, a decision was made based on mutual agreement.

Phase 2: narrative synthesis of measurement properties

The measurement properties of the identified tools were taken from the original development and validation studies using lateral search (if and when possible). The information, such as the author’s name, study population, and measurement properties, including internal consistency, reliability, measurement error, structural validity, hypothesis validity, cross-cultural validity, criterion validity, and responsiveness, was extracted.

Assessment of Risk of Bias for Measurement Properties

Using the COSMIN checklist, the authors (TK, NR, and AK) assessed the risk of bias for the measurement properties of instruments. As per the COSMIN checklist, the quality of instruments is categorized as very good, adequate, doubtful, or inadequate. The following steps were followed to evaluate the quality of the instrument using the COSMIN checklist: (i) identification of the measurement properties assessed in the study; (ii) understanding of statistical methods used for assessing the measurement properties; (iii) evaluation of the properties according to COSMIN-checklist items; and (iv) grading of each property on the basis of its quality (very good to inadequate). Finally, the methodological quality of a property (e.g., internal consistency) corresponded to the lowest rating given to an item in the COSMIN checklist for that section. For example, if an item on the COSMIN checklist for internal consistency was rated poor, whereas all other items were rated as good, then the final quality for internal consistency was classified as poor.

Results

Identification of MUPS Studies

**Figure 1:**
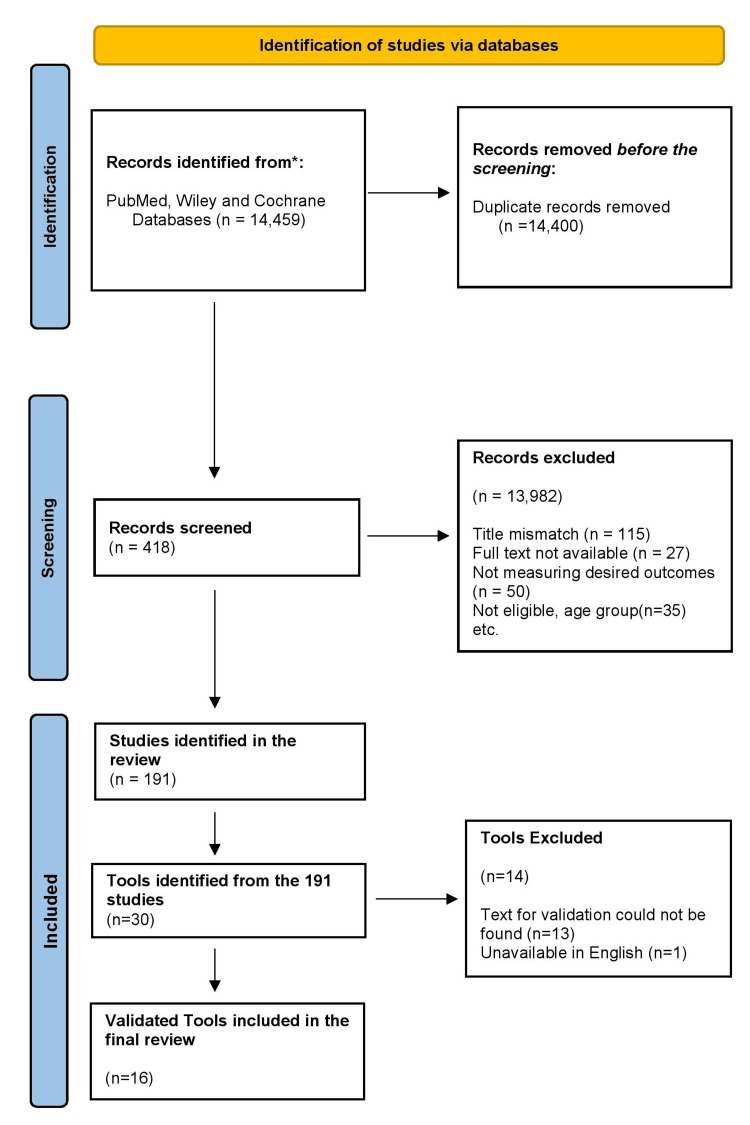
PRISMA flowchart PRISMA: Preferred Reporting Items for Systematic Reviews and Meta-Analyses

A total of 14,459 studies were identified in the initial search. Out of these, 191 studies were identified, as shown in the PRISMA flow chart (Figure 1). Studies were randomized controlled trials, observational, and intervention studies. The participant's age ranged from 18 to 60. The studies were conducted in psychiatric and medical settings.

Identification of MUPS Tools to Assess Symptoms and Symptom Severity

From 191 studies, a total of 16 validated tools were included, as presented in Table 1. These tools assessed the symptoms and symptom severity of patients diagnosed with MUPS.

**Table 1:** List of validated tools NA: not available, HVS: hyperventilation syndrome, CDC: Centers for Disease Control and Prevention, CFS: chronic fatigue syndrome, SCAN: Schedules for Clinical Assessment in Neuropsychiatry

S. No	Questionnaire	Study design	Age	Setting	Sample size	Country	Items	Domain assessed	Scale	Time frame (duration of persistent symptoms)	Target population	Multiple languages
1.	Patient Health Questionnaire-15 (PHQ-15) [5]	Cross-sectional	18 years or older	Primary care	Total: 3000 (1422 from five general internal medicine clinics and 1578 from three family practice clinics)	New York	15	Somatic symptom severity	3 categories: not bothered at all (0); bothered a little (1); bothered a lot (2)	Past 4 weeks	Patients; adults	Yes
2.	Somatic Symptom Scale-China (SSS-CN) [6]	Cross-sectional	30-60 years	Otorhinolaryngology department	N=883	Egypt	20	Somatic symptom severity	4 categories: from not existent to almost every day	From 6 months	Outpatients with SSD	
3.	Central Sensitization Inventory (CSI) [7]	Validation and reliability	NA	Patients	NA	NA	25	Intensity of somatic complaints	4 categories: not at all to a whole lot	Past two weeks	Patients; adults	No
4.	Brief Symptom Inventory (BSI) [8]	Survey	13-17 years	Both clinical and non-clinical samples of adolescents (Hispanic population)	N=1037	NA	53	Somatization	5 categories: not at all to always	Past week	Adolescents and adults	Yes
5.	Stress and Crisis Inventory-93 (SCI-93) [9]	Cross-sectional and comparative	18-60 years	Women	N=183	USA	35	Severity of symptoms that are primarily related to the autonomic nervous system	4 point scale	NA	Patients; adults	Yes
6.	Screening for Somatoform Disorders-7 T (SOMS-7 T) [10]	Correlation study	35-47 years	Disabled inpatients	N=324	Prien and Germany	53	Somatic symptom severity	5-point Likert scale (answering options range between 0 to 4)	Past week	Patients; adults	Yes
7.	ICD-10 Symptom List [11]	Link NA	NA	NA	NA	St. Louis	14	Somatization disorder	Yes/no	Past 2 years	Patients	Yes
8.	Nijmegen Hyperventilation List (NHL) [12]	Link NA	NA	HVS and non-HVS patients	N=263	Netherlands	16	Hyperventilation	5 categories from: never to very often	NA	Patients; adults	Yes
9.	Global Assessment of Functioning (GAF) Scale [13]	Review	NA	NA	38-40 articles	NA	18	Functional impairment (used to rate how serious a mental illness may be)	Scores range from 100 (extremely high functioning) to 1 (severely impaired)	NA	Patients; adults	Yes
10.	Short-Form Nepean Dyspepsia Index (SF-NDI) [14]	Correlational	English-speaking patients: 56.2 ± 14 and Malay-speaking patients: 43.3 ± 14.9	Outpatients with dyspepsia attending the gastroenterology clinic of the university	N=143	Malaysia	10	Functional dyspepsia	5-point Likert scale graded from 1 to 5	NA	Adults	Yes
11.	Checklist Individual Strength (CIS) [15]	Survey	>18 years	University, Radboud University Medical Center, tertiary treatment center, general population	Psychometric properties of the CIS (n=2288), general Dutch population (n=320), cancer survivors (n=1407), patients meeting CDC criteria for CFS, cut-off score for severe fatigue (n=5243), severely fatigued (n=1906), general population	Netherlands	20	Fatigue	7-point Likert scale	Past 2 weeks	Rescue workers and residents	Yes
12.	Short-form McGill Pain Questionnaire (SF-MPQ) [16]	Prospective	>60 years	Primary care	N=190	Canada	15	Pain	Category 4, from 0 to 3, none to severe	NA	Patients	Yes
13.	Bodily Distress Syndrome (BDS) Checklist [17]	Cohort	18-60 years	Primary care	N=492	Denmark	25	BDS; pattern of symptoms rather than a simple symptom count (based on SCAN interview)	5 categories: not at all to a lot	Past month	Patients; adults	Yes
14.	Somatosensory Amplification Scale (SSAS) [18]	Cohort	16-25 years	University setting	N=375	France	10	Physical discomfort	5-point Likert scale	NA	Patients; adults	Yes
15.	Quality of Life in Reflux and Dyspepsia (QOLRAD) Questionnaire [19]	Cross-sectional	>18 years	NA	N=142	Turkey	25	Symptoms of heartburn or acid regurgitation	7-point Likert scale	Past week	Patients; adults	Yes
16.	Four-Dimensional Symptom Questionnaire (4-DSQ) [20]	Longitudinal internet study	≥18 years	Mental health setting	Dutch sample (n=159), US sample (n=159)	US, Netherland	16	Somatization	5 categories: no to very often or constantly	Past week	Primary care patients	Yes

Somatic Symptoms

The most common tool to study somatic symptom severity was the Patient Health Questionnaire-15 (PHQ-15) [5]. It comprises 15 somatic symptoms, and the patient is asked to rate them from 0 (not bothered at all) to 2 (bothered a lot). The tool can be self-administered or administered in an interview format. The Bodily Distress Syndrome (BDS) Checklist [17] is another tool comprising 30 items that can be self-administered to screen for somatic symptoms. Other tools that assess the somatic symptoms or can be used as a screening tool for somatization are the Self-reported Somatic Symptom Scale-China (SSS-CN) [6], the Four-Dimensional Symptom Questionnaire (4-DSQ) [20], the Central Sensitization Inventory (CSI) [7], Screening for Somatoform Disorders-7 (SOMS-7 T) [10], the ICD-10 Symptom List [11], and the Somatosensory Amplification Scale (SSAS) [18].

The other components of somatic symptoms, such as pain, were assessed using a short-form McGill Pain Questionnaire (SF-MPQ) [16] and a psychological stress measure [21]. The clinical interview schedule [22] assesses the level of fatigue among MUPS patients. Symptoms of hyperventilation were assessed using Nijmegen Hyperventilation List (NHL) tools [12].

Measurement Properties of Instruments: A Narrative Synthesis

The primary development and validation studies were identified using the lateral search to look for the measurement properties of the instruments. Out of 30 tools identified, only 16 validated tools were included in this review, as the remaining 14 tools were either in some other language or the validation papers were not available. The eight properties, namely, internal consistency, reliability, structural validity, hypothesis validity, cross-cultural validity, responsiveness, criterion validity, and measurement error, were assessed. Information regarding the development and validation of only 16 somatic tools could be identified. The internal consistency was calculated using Cronbach’s alpha (CA). CA >0.70 was reported for all the somatic instruments. The reliability was reported as interclass correlation coefficients (ICC) or Pearson’s or Spearman’s correlation coefficients (r). For most of the tools, the ICC/r>0.70 except for the Brief Symptom Inventory (BSI) (r=0.68) [8]. These measurement properties have been presented in Table 2.

**Table 2:** Measurement properties of somatic symptom tools NE: not evaluated, α: alpha (or coefficient alpha) measures reliability or internal consistency, CFI: confirmatory fit index, TLI: Tucker-Lewis Index, RMSEA: root mean square error of approximation, AIC: Akaike information criterion, CI: confidence interval, SRMR: standardized root mean square residual, KMO: Kaiser-Meyer-Olkin, AUC: area under the curve, ROC: receiver operating curve, VUS: variant of uncertain significance, ICC: interclass correlation coefficients, CFA: confirmatory factor analysis, STAI Y: State-Trait Anxiety Inventory Form Y, SCL: Symptom Checklist, CA: Cronbach’s alpha

Studies	Measure	Study population	Measurement properties							
			Internal consistency (CA)	Reliability	Measurement error	Structural validity	Hypothesis validity	Cross-cultural Validity	Responsiveness	Criterion validity
1. Kroenke et al., 2002 [5]	Patient Health Questionnaire-15 (PHQ-15)	N=3000	Cronbach’s 𝛼: 0.80	NE	NE	NE	Primary care (r=0.10), obstetrics-gynecology (r=0.14)	NE	NE	NE
2. Jiang et al., 2018 [6]	Somatic Symptom Scale-China (SSS-CN)	N=852, A=1880	NE	Test-retest reliability: 0.9	NE	NE	The correlation between the SSS-CN and PHQ-15 scores was 0.6. With a non-inferiority margin of 0.05, α=0.025, and β=0.8	NE	NE	AUC of the ROC=0.05, α=0.025, VUS=0.1, α=0.025
3. Kleinstäuber et al., 2021 [20]	Four-Dimensional Symptom Questionnaire (4-DSQ)	N=159	Cronbach’s 𝛼: US sample: 0.91 (0.89, 0.93), Dutch sample: 0.92 (0.90, 0.93)	McDonald’s omega, US sample: 0.92 (0.90, 0.93), Dutch sample: 0.91 (0.89, 0.93)	CFI: 0.977, TLI: 0.974, RMSEA: 0.072, 90% CI: 0.061-0.084	NE	A large positive correlation (r>0.78) was found. Large correlation coefficients (0.56 ≥ r ≥0.66) were also found	NE	NE	OQ-45 symptom distress, r=0.66, p< 0.001, OQ-45, interpersonal relationship, r=0.35, p<0.001, OQ-45 social role, r=0.43, p<0.001, AUC: 0.671, p=0.028, cutoff of ≥9.5 had a sensitivity of 0.556 and a specificity of 0.742
4. Neblett et al., 2018 [7]	Central Sensitization Inventory (CSI)	N=268	Cronbach’s α: 0.88	Pearson’s r=0.82	NE	NE	NE	NE	NE	AUC and standard errors for the CSI=0.86 and 0.03, with a significance level of 0.05
5. Derogatis and Melisaratos, 1975 [8]	Brief Symptom Inventory (BSI)	N=1037, A: non-patient adults, adolescents aged 13-17, adult psychiatric outpatients, and adult psychiatric inpatients	NE	Test re-test reliability: 0.68	NE	NE	Correlations between BSI and Wiggins content scales and the Tryon cluster scores (MMPI) ranged from 0.30 to 0.72, correlations between the BSI and SCL-R-90 were 0.92 to 0.99	NE	NE	NE
6. Ericsson et al., 2015 [9]	The Stress and Crisis Inventory-93 (SCI-93)	N=40, A=22-60	Cronbach's α: 0.98	Test re-test reliability: 0.95 or 0.60 to 0.95 (ICC)	NE	Eigenvalue: 18.7, variance: 53.3%, latent extracted factor: 0.49-0.84	NE	NE	NE	NE
7. Rief and Hiller, 2003 [10]	Screening for Somatoform Disorders-7 T (SOMS-7 T)	N=307	Cronbach’s α: 0.92	Test-retest reliability, symptom count: 0.76, severity index: 0.71	NE	NE	The SOMS-7 variables revealed higher associations with somatization scores (such as SCL-90-R) than with depression or anxiety scores. SOMS-7 scores	NE	NE	Correlations of psychological measures; CC: 0.67-0.76, behavioral medicine clinic (differences in scores between admission and discharge for patients); CC: 0.21-0.41
8. Janca et al., 1993 [11]	ICD-10 Symptom List	N=30, A=20-60	NE	Overall Kappa: 0.72	NE	NE	NE	NE	NE	NE
9. van Dixhoorn et al., 2015 [12]	Nijmegen Hyperventilation List (NHL)	N=162, A=20-62	Cronbach’s α: 0.92 (single-factor model	Inter-rater: 0.98	NE	NE	KMO: 0.926; single-factor model, with 58.6% of explained variability, convergent validity: g (r=0.81, p<0.001)	NE	NQ correlated with ETCO2 (r=-0.68), RR (r=-0.66), and BHT (r=-0.65)	AUC=0.05, 92.73% sensitivity and 91.59% specificity
10. Aas., 2010 [13]	Global Assessment of Functioning (GAF) Scale	NE	NE	NE	NE	NE	GAF-S and GAF-F can be correlated with r=0.61	NE	NE	NE
11. Mahadeva et al., 2009 [14]	Short-Form Nepean Dyspepsia Index (SF-NDI)	N=143	α values ranging from 0.83 to 0.88 (English) and from 0.83 to 0.90 (Malay)	0.90 (English) and 0.83 (Malay)	NE	NE	Good correlation (r=0.3-0.6) between all SF-NDI sub-scales and various domains of the SF-36	NE	NE	NE
12. Worm-Smeitinka et al., 2017 [15]	Checklist Individual Strength (CIS)	N=2288	Internal consistency (α=0.84–0.95), Cronbach’s α: 0.95, (fatigue severity: 0.94; concentration: 0.89; motivation: 0.84; activity: 0.90)	Test-retest reliability (r=0.74-0.86)	NE	Correlation coefficients >0.3, Eigenvalue of >1, 52.0%, 8.8%, 7.5%, and 5.2% of the variance, respectively (total variance 73.4%)	Correlations of the CIS (subscales) with the (sub)scales of the alternative measures should be high (>0.6)	NE	NE	ROC: 0.98 (95% CI: 0.977-0.983)
13. Gauthier et al., 2014 [16]	Short-form McGill Pain Questionnaire (SF-MPQ)	N=244	χ2(1)=4.06, p=0.04	NE	SRMR: 0.10, RMSEA: 0.07, CFI: 0.76, TLI: 0.75, AIC: 970.44	NE	NE	NE	NE	Total score was no longer significantly stronger in older than younger patients (α=0.92 vs. α=0.89, p=0.13)
14. Petersen et al, 2020 [17]	Bodily Distress Syndrome (BDS) Checklist	N=9656, A=18-69	Internal consistency was high (α≥0.879), general population α=0.887, primary care α=0.908, and specialized setting α=0.879	NE	RMSEA one-level one-factor CFA, general population: 0.111, 0.062, primary care: 0.419, 0.082, specialized setting: 0.149, 0.091, two-level four-factor CFA, general population: 0.061, primary care: 0.08, specialized setting: 0.089, bifactor CFA, general population: 0.048, primary care: 0.053, specialized setting: 0.059	NE	NE	NE	Response rate: 33.7%	Convergent validity of overall health (r=0.25-0.58), physical functioning (r=0.22-0.58), emotional distress (r=0.47-0.62), and illness worry (r=0.36-0.55)
15. Bridou et al., 2012 [18]	Somatosensory Amplification Scale (SSAS)	N=375	Chronbach's α: 0.81	NE	1-factor model CFI: 0.93, SRMR: 0.048, RMSEA 0.06, RMSEA 90% CI: 0.05-0.09, 2-factor model CFI: 0.83, SRMR: 0.161, RMSEA: 0.09, RMSEA 90% CI: 0.08-0.11	NE	SCL-90-R somatization subscale and STAI Y form (r=0.21, p<0.05)	NE	NE	NE
16. Hançerlioğlu et al., 2019 [19]	Quality of Life in Reflux and Dyspepsia (QOLRAD) Questionnaire	N=142, A>18	Cronbach’s α: 0.97	ICC: 0.97 (vitality) and 0.99 (eating/drinking disorders)	NE	Lowest correlation (emotional distress and role-emotional limitations): 0.10, highest correlation (between energy and vitality): 0.34	NE	NE	NE	NE

Risk of Bias Assessment of Instruments: A Quality Assessment

Most of the tools assessing the somatic symptoms had very good internal consistency and structural validity. The reliability of the majority of the tools was found to be adequate. Approximately half of the tools revealed very good convergent validity. However, cross-cultural validity was not reported for the majority of the tools. A detailed description of the tools is presented in the Appendices section.

Discussion

This systematic review identifies the instruments used in the literature to assess the symptoms and symptom severity among patients of MUPS and provides a narrative synthesis of their measurement properties. The standardized tools may help clinicians and researchers to generate data across diverse population groups and draw reliable conclusions.

MUPS patients are presented with somatic complaints varying from mild to severe. Patients with such symptoms constantly juggle from one physician to another, contributing to the overutilization of healthcare services. In literature, instruments for the assessment of somatic symptoms and their severity have been defined as generic (somatic complaints or somatic symptom severity) or condition-specific (dyspepsia, pain, fatigue). Patient Health Questionnaire-15 (PHQ-15) is the most widely used tool to assess somatization or somatic severity. The instrument is highly valid and reliable and can be self-administered. However, in most cases, the tool is often coupled with other psychiatric instruments to capture the greater aspect of the problem.

The Stress and Crisis Inventory-93 (SCI-93) [9] can assess the severity of stress symptoms related to the autonomic nervous system. This self-reported tool can also assess symptoms due to the impact of trauma. The tool has high internal consistency and structural, convergent, and criterion validity.

The pain and physical discomfort among MUPS patients can be evaluated using Somatosensory Amplification Scale (SSAS). The instrument helps in assessing bodily sensations. The three components gauged using this tool are body hypervigilance, rare and weak body sensations, and emotional reaction to the sensations [18]. The tool is easy to self-administer and has extremely well-established validity and reliability. Lastly, the Checklist Individual Strength (CIS) is a reliable, valid, and easy-to-administer tool for assessing fatigue severity, concentration problems, and reduced motivation.

All the above-stated tools are self-administered and have been developed on a Likert scale. They can be used to capture the overall somatic domain in MUPS patients as they depict very good psychometric properties.

Recent studies from India highlight the unique perspectives of patients, caregivers, and healthcare professionals regarding MUPS [23]. These insights underscore the necessity for locally tailored evaluation modules, recognizing the distinct symptom presentations in Indian populations compared to the West [24,25]. In response, certain researchers have formulated comprehensive treatment modules, coupled with yoga therapy, to address the needs of these patients effectively [26,27].

Limitations and strengths

Some limitations of the present review should be considered. We evaluated only three databases through electronic searches. We did not look for unpublished data or other algorithmic search engines like Google Scholar. We limited ourselves to studies from 2000 onwards, though there may have been some studies on the topic before 2000. We did not delve into different sub-populations that presented with MUPS, such as adolescents and geriatric populations. Lastly, we considered somatic symptoms to be synonymous with MUPS. Despite the limitations, the systematic assessment and narrative synthesis of the tools may be a handy resource for researchers and clinicians working in the field of MUPS.

Recommendation for future research

This review found that most of the tools identified have not been cross-culturally validated. The symptoms of MUPS may differ from culture to culture, as patients provide their perception or meaning to their symptoms. Therefore, either these tools should be validated culturally before administering, or a tool should be developed and validated according to the symptoms presented in a particular cultural setting.

## Conclusions

A systematic review has been done to identify the validated tools used to assess the symptoms and symptom severity in MUPS patients. A narrative synthesis has also been performed to assess the methodological quality of the tools. A bird-eye view of the standardized tools has been provided, which can be used by clinicians and public health researchers for the assessment of MUPS patients at baseline and to assess the effectiveness and efficacy of treatment to improve their physical and psychological well-being.

## Appendices

**Table 3:** Risk of bias assessment of instruments (reliability) NA: not available

Study	Instrument	Internal consistency			Reliability				
		Full scale or subscale	Statistical method	Final decision	Patient stable	Adequate time interval	Similar test condition	Statistical test	Final decision
1. Kroenke et al., 2002 [5]	Patient Health Questionnaire-15 (PHQ-15)	v good	v good	v good	NA	NA	NA	NA	NA
2. Jiang et al., 2018 [6]	Somatic Symptom Scale-China (SSS-CN)	NA	NA	NA	doubtful	doubtful	doubtful	v good	adequate
3. Kleinstäuber et al., 2021 [20]	Four-Dimensional Symptom Questionnaire (4-DSQ)	v good	v good	v good	doubtful	doubtful	doubtful	v good	adequate
4. Neblett et al., 2018 [7]	Central Sensitization Inventory (CSI)	v good	v good	v good	doubtful	doubtful	doubtful	v good	adequate
5. Derogatis and Melisaratos, 1975 [8]	Brief Symptom Inventory (BSI)	NA	NA	NA	doubtful	doubtful	doubtful	v good	adequate
6. Ericsson et al., 2015 [9]	The Stress and Crisis Inventory-93 (SCI-93)	v good	v good	v good	adequate	adequate	doubtful	v good	adequate
7. Rief and Hiller, 2003 [10]	Screening for Somatoform Disorders-7 T (SOMS-7 T)	v good	v good	v good	doubtful	doubtful	doubtful	v good	adequate
8. Janca et al., 1993 [11]	ICD-10 Symptom List	NA	NA	NA	doubtful	doubtful	doubtful	v good	adequate
9. van Dixhoorn et al., 2015 [12]	Nijmegen Hyperventilation List (NHL)	v good	v good	v good	adequate	adequate	adequate	v good	adequate
10. Aas., 2010 [13]	Global Assessment of Functioning (GAF) Scale	NA	NA	NA	NA	NA	NA	NA	NA
11. Mahadeva et al., 2009 [14]	Short-Form Nepean Dyspepsia Index (SF-NDI)	v good	v good	v good	doubtful	v good	v good	v good	v good
12. Worm-Smeitinka et al., 2017 [15]	Checklist Individual Strength (CIS)	v good	v good	v good	doubtful	v good	doubtful	v good	adequate
13. Gauthier et al., 2014 [16]	Short-form McGill Pain Questionnaire (SF-MPQ)	v good	v good	v good	doubtful	doubtful	doubtful	v good	adequate
14. Petersen et al, 2020 [17]	Bodily Distress Syndrome (BDS) Checklist	v good	v good	v good	NA	NA	NA	NA	NA
15. Bridou et al., 2012 [18]	Somatosensory Amplification Scale (SSAS)	v good	v good	v good	NA	NA	NA	NA	NA
16. Hançerlioğlu et al., 2019 [19]	Quality of Life in Reflux and Dyspepsia (QOLRAD) Questionnaire	v good	v good	v good	doubtful	v good	doubtful	v good	adequate

**Table 4:** Risk of bias assessment of instruments (structural and cross-cultural validity) EFA: exploratory factor analysis, CFA: confirmatory factor analysis, NA: not available

Study	Instrument	Structural validity					Cross-cultural validity			
		EFA/CFA	Chosen model and research question	Sample size	Methodological flaws	Final decision	Similar sample	Data analysis	Sample size	Final decision
1. Kroenke et al., 2002 [5]	Patient Health Questionnaire-15 (PHQ-15)	v good	doubtful	v good	v good	v good	NA	NA	NA	NA
2. Jiang et al., 2018 [6]	Somatic Symptom Scale-China (SSS-CN)	v good	doubtful	v good	v good	v good	NA	NA	NA	NA
3. Kleinstäuber et al., 2021 [20]	Four-Dimensional Symptom Questionnaire (4-DSQ)	v good	doubtful	doubtful	v good	v good	v good	v good	v good	v good
4. Neblett et al., 2018 [7]	Central Sensitization Inventory (CSI)	adequate	v good	v good	v good	v good	v good	v good	v good	v good
5. Derogatis and Melisaratos, 1975 [8]	Brief Symptom Inventory (BSI)	Inadequate	v good	v good	v good	v good	NA	NA	NA	NA
6. Ericsson et al., 2015 [9]	The Stress and Crisis Inventory-93 (SCI-93)	v good	adequate	v good	v good	v good	NA	NA	NA	adequate
7. Rief and Hiller, 2003 [10]	Screening for Somatoform Disorders-7 T (SOMS-7 T)	adequate	adequate	inadequate	v good	adequate	NA	NA	NA	adequate
8. Janca et al., 1993 [11]	ICD-10 Symptom List	NA	NA	NA	NA	NA	adequate	adequate	v good	adequate
9. van Dixhoorn et al., 2015 [12]	Nijmegen Hyperventilation List (NHL)	NA	NA	NA	NA	NA	NA	NA	NA	NA
10. Aas., 2010 [13]	Global Assessment of Functioning (GAF) Scale	NA	NA	NA	NA	NA	NA	NA	NA	NA
11. Mahadeva et al., 2009 [14]	Short-Form Nepean Dyspepsia Index (SF-NDI)	NA	NA	NA	NA	NA	v good	v good	v good	v good
12. Worm-Smeitinka et al., 2017 [15]	Checklist Individual Strength (CIS)	v good	v good	v good	v good	v good	NA	NA	NA	adequate
13. Gauthier et al., 2014 [16]	Short-form McGill Pain Questionnaire (SF-MPQ)	v good	v good	v good	v good	v good	NA	NA	NA	adequate
14. Petersen et al, 2020 [17]	Bodily Distress Syndrome (BDS) Checklist	v good	v good	v good	v good	v good	NA	NA	NA	adequate
15. Bridou et al., 2012 [18]	Somatosensory Amplification Scale (SSAS)	v good	v good	v good	v good	v good	NA	NA	NA	adequate
16. Hançerlioğlu et al., 2019 [19]	Quality of Life in Reflux and Dyspepsia (QOLRAD) Questionnaire	adequate	adequate	v good	NA	adequate	NA	NA	NA	adequate

**Table 5:** Risk of bias assessment of instruments (construct validity) NA: not available

Study	Instrument	Hypothesis testing of construct validity						
		Convergent validity				Discriminant validity		
		Comparator group measures	Sufficient measurement prop of comparator	Statistical measurement	Final decision	Characteristic of sub-group	Statistical method	Final decision
1. Kroenke et al., 2002 [5]	Patient Health Questionnaire-15 (PHQ-15)	v good	v good	v good	v good	inadequate	inadequate	inadequate
2. Jiang et al., 2018 [6]	Somatic Symptom Scale-China (SSS-CN)	NA	NA	NA	NA	NA	NA	NA
3. Kleinstäuber et al., 2021 [20]	Four-Dimensional Symptom Questionnaire (4-DSQ)	adequate	adequate	v good	adequate	adequate	v good	adequate
4. Neblett et al., 2018 [7]	Central Sensitization Inventory (CSI)	v good	v good	v good	v good	NA	NA	NA
5. Derogatis and Melisaratos, 1975 [8]	Brief Symptom Inventory (BSI)	NA	NA	NA	NA	NA	NA	NA
6. Ericsson et al., 2015 [9]	The Stress and Crisis Inventory-93 (SCI-93)	v good	doubtful	v good	v good	NA	NA	NA
7. Rief and Hiller, 2003 [10]	Screening for Somatoform Disorders-7 T (SOMS-7 T)	v good	v good	v good	v good	NA	NA	NA
8. Janca et al., 1993 [11]	ICD-10 Symptom List	v good	v good	v good	v good	NA	NA	NA
9. van Dixhoorn et al., 2015 [12]	Nijmegen Hyperventilation List (NHL)	NA	NA	NA	NA	NA	NA	NA
10. Aas., 2010 [13]	Global Assessment of Functioning (GAF) Scale	NA	NA	NA	NA	NA	NA	NA
11. Mahadeva et al., 2009 [14]	Short-Form Nepean Dyspepsia Index (SF-NDI)	v good	v good	v good	v good	NA	NA	NA
12. Worm-Smeitinka et al., 2017 [15]	Checklist Individual Strength (CIS)	NA	NA	NA	NA	v good	v good	v good
13. Gauthier et al., 2014 [16]	Short-form McGill Pain Questionnaire (SF-MPQ)	v good	v good	v good	v good	v good	v good	v good
14. Petersen et al, 2020 [17]	Bodily Distress Syndrome (BDS) Checklist	NA	NA	NA	NA	NA	NA	NA
15. Bridou et al., 2012 [18]	Somatosensory Amplification Scale (SSAS)	v good	v good	v good	v good	v good	v good	v good
16. Hançerlioğlu et al., 2019 [19]	Quality of Life in Reflux and Dyspepsia (QOLRAD) Questionnaire	v good	v good	v good	v good	NA	NA	NA

**Table 6:** Risk of bias assessment of instruments (criterion validity) NA: not available

Study	Instrument	Criterion validity					
		Statistical method	Other	Final decision	Design required	Statistical measures	Final decision
1. Kroenke et al., 2002 [5]	Patient Health Questionnaire-15 (PHQ-15)	NA	NA	NA	NA	NA	NA
2. Jiang et al., 2018 [6]	Somatic Symptom Scale-China (SSS-CN)	v good	doubtful	v good	NA	NA	NA
3. Kleinstäuber et al., 2021 [20]	Four-Dimensional Symptom Questionnaire (4-DSQ)	v good	v good	v good	NA	NA	NA
4. Neblett et al., 2018 [7]	Central Sensitization Inventory (CSI)	v good	doubtful	v good	v good	v good	v good
5. Derogatis and Melisaratos, 1975 [8]	Brief Symptom Inventory (BSI)	NA	NA	NA	NA	NA	NA
6. Ericsson et al., 2015 [9]	Stress and Crisis Inventory-93 (SCI-93)	v good	doubtful	v good	NA	NA	NA
7. Rief and Hiller, 2003 [10]	Screening for Somatoform Disorders-7 T (SOMS-7 T)	v good	v good	v good	v good	v good	v good
8. Janca et al., 1993 [11]	ICD-10 Symptom List	v good	v good	v good	v good	v good	v good
9. van Dixhoorn et al., 2015 [12]	Nijmegen Hyperventilation List (NHL)	v good	v good	v good	v good	v good	v good
10. Aas., 2010 [13]	Global Assessment of Functioning (GAF) Scale	v good	v good	v good	NA	NA	NA
11. Mahadeva et al., 2009 [14]	Short-Form Nepean Dyspepsia Index (SF-NDI)	NA	NA	NA	NA	NA	NA
12. Worm-Smeitinka et al., 2017 [15]	Checklist Individual Strength (CIS)	v good	v good	v good	v good	v good	v good
13. Gauthier et al., 2014 [16]	Short-form McGill Pain Questionnaire (SF-MPQ)	v good	v good	v good	v good	v good	v good
14. Petersen et al., 2020 [17]	Bodily Distress Syndrome (BDS) Checklist	v good	doubtful	v good	NA	NA	NA
15. Bridou et al., 2012 [18]	Somatosensory Amplification Scale (SSAS)	v good	v good	v good	NA	NA	NA
16. Hançerlioğlu et al., 2019 [19]	Quality of Life in Reflux and Dyspepsia (QOLRAD) Questionnaire	NA	NA	NA	NA	NA	NA

**Table 7:** Risk of bias assessment of instruments (responsiveness) NA: not available

Study	Instrument	Responsiveness (intervention)								Responsiveness (measurement instrument)				
		Design required	Statistical measures	Final decision	Comparator instrument measure	Sufficient measurement	Statistical method	Other	Final decision	Comparator instrument measure	Sufficient measurement	Statistical method	Other	Final decision
1. Kroenke et al., 2002 [5]	Patient Health Questionnaire-15 (PHQ-15)	NA	NA	NA	NA	NA	NA	NA	NA	NA	NA	NA	NA	NA
2. Jiang et al., 2018 [6]	Somatic Symptom Scale-China (SSS-CN)	NA	NA	NA	v good	v good	v good	doubtful	v good	v good	v good	v good	doubtful	v good
3. Kleinstäuber et al., 2021 [20]	Four-Dimensional Symptom Questionnaire (4-DSQ)	NA	NA	NA	v good	adequate	v good	doubtful	adequate	v good	adequate	v good	doubtful	adequate
4. Neblett et al., 2018 [7]	Central Sensitization Inventory (CSI)	v good	v good	v good	v good	v good	v good	v good	v good	v good	v good	v good	v good	v good
5. Derogatis and Melisaratos, 1975 [8]	Brief Symptom Inventory (BSI)	NA	NA	NA	NA	NA	NA	NA	NA	NA	NA	NA	NA	NA
6. Ericsson et al., 2015 [9]	The Stress and Crisis Inventory-93 (SCI-93)	NA	NA	NA	NA	NA	NA	NA	NA	NA	NA	NA	NA	NA
7. Rief and Hiller, 2003 [10]	Screening for Somatoform Disorders-7 T (SOMS-7 T)	v good	v good	v good	v good	v good	v good	v good	v good	v good	v good	v good	v good	v good
8. Janca et al., 1993 [11]	ICD-10 Symptom List	v good	v good	v good	v good	v good	v good	v good	v good	v good	v good	v good	v good	v good
9. van Dixhoorn et al., 2015 [12]	Nijmegen Hyperventilation List (NHL)	v good	v good	v good	v good	v good	v good	v good	v good	v good	v good	v good	v good	v good
10. Aas., 2010 [13]	Global Assessment of Functioning (GAF) scale	NA	NA	NA	NA	NA	NA	NA	NA	NA	NA	NA	NA	NA
11. Mahadeva et al., 2009 [14]	Short-Form Nepean Dyspepsia Index (SF-NDI)	NA	NA	NA	v good	v good	v good	v good	v good	v good	v good	v good	v good	v good
12. Worm-Smeitinka et al., 2017 [15]	Checklist Individual Strength (CIS)	v good	v good	v good	v good	v good	v good	v good	v good	v good	v good	v good	v good	v good
13. Gauthier et al., 2014 [16]	Short-form McGill Pain Questionnaire (SF-MPQ)	v good	v good	v good	v good	v good	v good	v good	v good	v good	v good	v good	v good	v good
14. Petersen et al, 2020 [17]	Bodily Distress Syndrome (BDS) Checklist	NA	NA	NA	NA	NA	NA	NA	NA	NA	NA	NA	NA	NA
15. Bridou et al., 2012 [18]	Somatosensory Amplification Scale (SSAS)	NA	NA	NA	v good	v good	v good	v good	v good	v good	v good	v good	v good	v good
16. Hançerlioğlu et al., 2019 [19]	Quality of Life in Reflux and Dyspepsia (QOLRAD) Questionnaire	NA	NA	NA	NA	NA	NA	NA	NA	NA	NA	NA	NA	NA
